# Associations Among Handgrip Strength, Brain Pathology, and Cognition in Autosomal Dominant Alzheimer’s disease

**DOI:** 10.1093/geroni/igaf122.3544

**Published:** 2025-12-31

**Authors:** Nadeshka Ramirez-Perez, Ana Baena, Daniel Vasquez, Lusiana Martinez, Averi Giudicessi, Liliana Ramirez Gomez, David Aguillon, Yakeel Quiroz

**Affiliations:** Massachusets General Hospital, Boston, Massachusetts, United States; Universidad de Antioquia, Medellin, Antioquia, Colombia; Universidad de Antioquia, Medellin, Antioquia, Colombia; Massachusets General Hospital, Boston, Massachusetts, United States; Massachusetts General Hospital, Boston, Massachusetts, United States; Massachusetts General Hospital, Boston, Massachusetts, United States; Massachusetts General Hospital, Boston, Massachusetts, United States; Boston University, Boston, Massachusetts, United States

## Abstract

**Objective:**

Grip strength decline, and cognitive impairment are prevalent in aging and may reflect shared underlying biological processes. This study examined associations between handgrip strength, brain pathology and cognition in individuals with/without a PSEN1 E280A mutation for autosomal dominant Alzheimer’s disease (ADAD). We investigated whether handgrip strength differed by mutation status and its association with age and tau pathology.

**Methods:**

52 participants from the Colombia-Boston Biomarker Study (COLBOS) (28 PSEN1-E280A carriers, 24 non-carriers; MAge 34.1 ± 5.5 years; MEducation =9.96 ± 3.94 years; 57.8% female) completed the Mini-Mental State Examination (MMSE), CERAD Word List Memory Test, handgrip test via dynamometer, and MRI and PET imaging for amyloid and tau pathology. Group differences were assessed using Mann–Whitney U tests. Spearman correlations explored associations between handgrip strength, cognition, and PET biomarkers.

**Results:**

Handgrip strength did not differ between carriers and non-carriers (Carriers: 74.44 +/- 35.34; non-carriers: 75.53 +/- 26.82; p = .62). Carriers had lower MMSE scores (U = 183.50, p = .004) and greater tau PET burden in the entorhinal cortex (p = .002–.037). Handgrip strength was unrelated to age, cognition or amyloid. Lower handgrip strength was significantly associated with greater tau burden in precuneus in the left hemisphere (ρ = –0.560, p = .019).

**Conclusion:**

Although handgrip strength was not linked to carrier status, cognition or amyloid, lower grip strength was associated with greater tau accumulation in the precuneus. These findings highlight handgrip strength as a potential low-cost marker of tau-related neurodegeneration in ADAD. Larger longitudinal studies are needed to extend these results.

